# Incidental Right Hilar Metallic Density After Implantable Cardioverter Defibrillator Placement in a Tracheostomized Woman: Suspected Airway Foreign Body With Concurrent Pleural Effusion

**DOI:** 10.7759/cureus.101866

**Published:** 2026-01-19

**Authors:** Michael J Martinez, Hardik Jain, Neeraj Goindani, Ahmed Bux, Anusha Gupta, Tanya Zagoruychenko

**Affiliations:** 1 Internal Medicine, St. Matthews University, Cayman Islands, CYM; 2 Internal Medicine, Brookdale University Hospital Medical Center, Brooklyn, USA

**Keywords:** airway foreign body, basilar atelectasis, flexible bronchoscopy, implantable cardioverter defibrillator, pleural effusion, portable chest radiograph, radiographic device artifact, right main bronchus, tracheostomy cleaning brush, tracheostomy complications

## Abstract

Adult tracheobronchial foreign bodies are uncommon and can be subtle on radiographs, particularly in device-rich environments. An 80-year-old woman with severe heart failure underwent elective implantable cardioverter defibrillator (ICD) placement and had a linear right hilar radiopaque density and right basilar atelectasis/pleural effusion on postoperative portable chest radiography. She was stable on a tracheostomy collar without respiratory distress. Given the appearance and history of home tracheostomy care, a tracheostomy cleaning brush was suspected. Flexible bronchoscopy via the tracheostomy confirmed and retrieved a cleaning brush from the right bronchus intermedius; purulent secretions were irrigated, and there were no complications. The patient remained hemodynamically stable post-procedure. This case illustrates the value of correlating imaging with clinical stability yet proceeding to definitive airway evaluation when suspicion persists, and it highlights tracheostomy-care equipment as a potential source of iatrogenic foreign bodies in adults.

## Introduction

Foreign body aspiration in adults is less common than in children and may present insidiously; bronchoscopy remains the diagnostic and therapeutic standard [1,2]. Cleaning brushes and other tracheostomy components are known to be iatrogenic foreign bodies in adults and are frequently radiopaque, allowing for radiographic detection and bronchoscopic removal [3,4]. However, radiographic identification is complicated in perioperative and ICU settings. Portable radiographs are prone to artifacts from monitoring leads and external devices that can mimic endobronchial material; careful device mapping and cross-sectional correlation help avoid misinterpretation [5]. Furthermore, foreign bodies may be radiolucent, though metallic iatrogenic objects are exceptions [6,7].

In complex cases, unilateral pleural effusions may coexist with atelectasis or aspiration. Guideline-based evaluation incorporates imaging and, when indicated, diagnostic thoracentesis to clarify etiology [8]. Interpreting pleural fluid results further guides management, including when to apply laboratory criteria during thoracentesis [9,10]. Computed tomography can help differentiate pleural effusion from parenchymal opacity and clarify confusing projections [11,12]. This case illustrates the value of correlating imaging with clinical stability yet proceeding to definitive airway evaluation when suspicion persists.

## Case presentation

An 80-year-old woman with hypertension, hyperlipidemia, type 2 diabetes (HbA1c 6.4% four months prior), obesity (BMI 31.2), chronic systolic heart failure (left ventricular ejection fraction or LVEF 30-35% with dilated inferior vena cava (IVC) and trace pericardial effusion), tracheostomy collar, and percutaneous endoscopic gastrostomy (PEG) tube presented on July 7, 2025 for elective implantable cardioverter defibrillator (ICD) implantation. Additional history included a recent left parietal convexity subdural hematoma and C1 vertebral fractures status post laminectomy. The ICD procedure was uncomplicated with an appropriate right ventricular lead position.

She was afebrile and hemodynamically stable post-procedure (blood pressure 158/82 → 151/87 mmHg (normal value: <120/80 mmHg); heart rate 70-82 bpm (normal values: 60-100 bpm); respiratory rate 18-20 breaths/min (normal values: 12-20 breaths/min); temperature 36.2-37.1 °C (normal values: 36.5°C-37.3°C); SpO₂ 97-100% (normal values: 95-100%) on tracheostomy collar ~35% FiO₂); comfortable, and without dyspnea or chest pain. 

Medications

The patient's medications and their dosing frequency are outlined in Table 1. 

**Table 1:** Current medications and their regimen Deep vein thrombosis (DVT) prophylaxis was managed with sequential compression devices (SCDs); chemical prophylaxis was initially held due to the recent history of subdural hematoma but was planned pending stability of coagulation profiles. The patient was placed on a cardiac diet (low sodium, low cholesterol) and bedrest with bathroom privileges. BID: Bis in die (twice a day); PO: Per os (by mouth); GT: Gastrostomy tube; Q4H: Every four hours; PRN: Pro re nata (as needed); U/g: Units per gram.

Medication	Dosage	Frequency	Route
Carvedilol	3.125 mg	BID	PO/GT
Sacubitril/Valsartan	24-26 mg	BID	PO/GT
Spironolactone	12.5 mg	Daily	PO/GT
Furosemide	20 mg	Daily	PO/GT
Dapagliflozin	10 mg	Daily	PO/GT
Acetylcysteine	200 mg/mL	Q4H	Nebulizer
Sodium chloride	0.90%	Q4H	Nebulizer
Omeprazole	20 mg	Daily	PO/GT
Polyethylene glycol	17 g	Daily	PO/GT
Multivitamin	1 tab	Daily	PO/GT
Vitamin B-12	1000 mcg	Daily	PO/GT
Ibuprofen	400 mg	PRN	PO/GT
Nystatin	100,000 U/g	BID	Topical

Exam

Alert and oriented; trach collar in place; no respiratory distress; lungs with normal breath sounds; regular cardiac rhythm without murmurs; soft, nondistended abdomen; no leg edema.

Laboratory data

The results of the laboratory parameters are given in Table 2.

**Table 2:** Laboratory results on admission The results demonstrated stable hematologic, renal, and hepatic parameters. All values were within or near expected limits for this patient’s postoperative and chronic comorbidity profile. WBC: White blood cell; BUN: Blood urea nitrogen; AST: Aspartate aminotransferase; ALT: Alanine aminotransferase; INR: International normalized ratio; POC: Point of care.

Parameter	Result	Reference range and units
Hemoglobin	10.5	12-15.5 g/dL
Hematocrit	31	36-46 %
WBC count	4.2	4.0-10.5 ×10^3^/µL
Platelets	239–263	150-400 ×10^3^/µL
Sodium	141	135-145 mmol/L
Potassium	3.6	3.5-5.0 mmol/L
Bicarbonate (HCO₃)	30	22-28 mmol/L
BUN	8.6	7-20 mg/dL
Creatinine	0.5	0.6-1.3 mg/dL
AST	21	10-40 U/L
ALT	12	7-56 U/L
Albumin	4.7	3.5-5.0 g/dL
INR	1.05	0.9-1.1
Glucose (POC)	168	70-140 mg/dL

Cardiac testing

**Figure 1:**
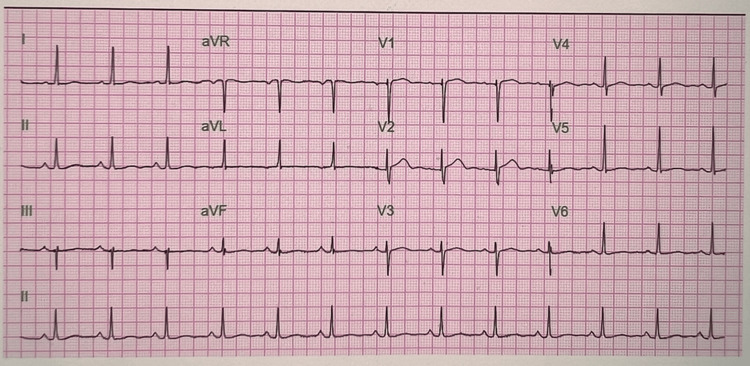
Twelve-lead ECG demonstrating normal sinus rhythm with nonspecific T-wave abnormalities Heart rate 77 bpm; PR 186 ms; QRS 76 ms; QTc 441 ms.

Twelve-lead ECG (performed on July 7, 2025) showed normal sinus rhythm (77 bpm) with nonspecific T-wave changes (PR 186 ms, QRS 76 ms, QTc 441 ms) (Figure 1).

Imaging

**Figure 2:**
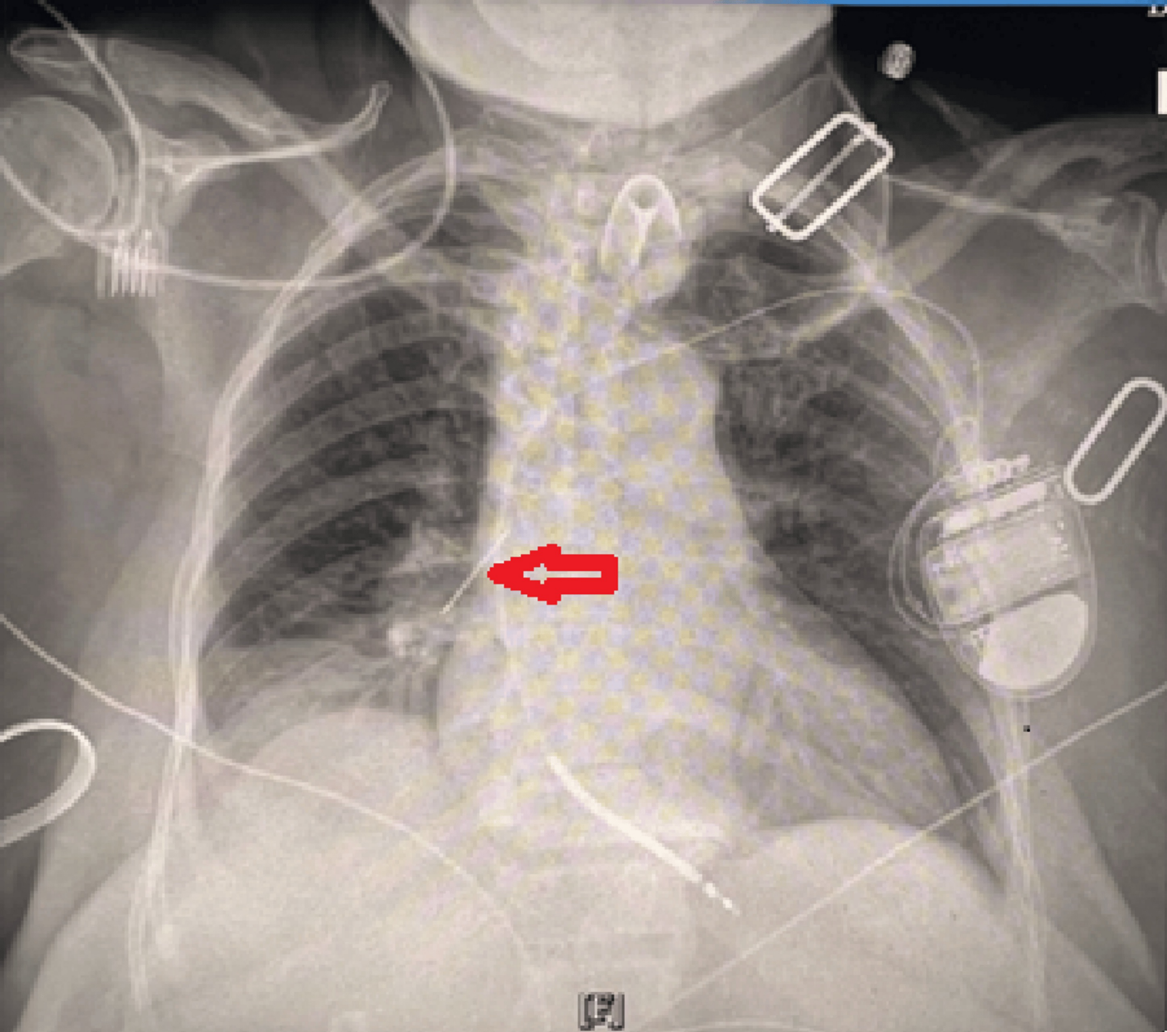
Portable AP chest radiograph showing left-sided ICD generator and right ventricular lead ICD: Implantable cardioverter defibrillator; A thin linear radiopaque density projects over the right hilum, with right basilar atelectasis and a small right pleural effusion.

Portable AP chest radiograph (performed on August 7, 2025) showed a left-sided ICD generator with transvenous lead to the right ventricle, a thin linear radiopaque density projected over the right pulmonary hilum (also present during an earlier chest radiograph taken shortly after the patient's admission but prior to the elective ICD placement), right basilar atelectasis with interval improvement, and a right pleural effusion. The left lung was clear (Figure 2).

Additional history

The patient’s daughter reported that a swivel tracheostomy cleaning brush had been used at home by a visiting nurse approximately two weeks before admission [13]. This history and the radiographic appearance increased concern for an intraluminal foreign body.

Procedure

On October 7, 2025, the thoracic surgery team performed flexible fiberoptic bronchoscopy through the existing tracheostomy site while the patient was under general anesthesia. The patient was categorized as American Society of Anesthesiologists (ASA) Physical Status Classification Class 3 [13], indicating a patient with severe systemic disease. A shoulder roll was used for neck extension. After timeout, a foreign body was visualized in the right bronchus intermedius. A snare was passed through the bronchoscope, the object was captured, and because it could not be fully withdrawn into the tracheostomy tube, the trach tube was removed. The foreign body was extracted and the trach was replaced easily. Bronchoscopy was repeated; purulent secretions were suctioned and the airway irrigated with sterile saline. No bleeding occurred. Estimated blood loss was minimal; intake recorded was lactated Ringer’s 50 mL and propofol 3 mL (total 53 mL).

The patient was transported to the post-anesthesia care unit (PACU) hemodynamically stable and a postoperative chest radiograph was ordered. The specimen (“foreign body right bronchus intermedius”) was sent to surgical pathology. It was wound class: IV [14]. There were no intraoperative complications. The operating room personnel were present per record and names have been omitted for privacy.

Outcome

The patient remained stable after bronchoscopy with no immediate complications.

## Discussion

Evaluation of suspected adult airway foreign bodies should begin by correlating imaging with clinical stability. While many aspirated foreign bodies are radiolucent, making diagnosis challenging [6], iatrogenic objects such as tracheostomy brushes are often metallic and radiopaque [3,4]. In this case, the portable chest radiograph revealed a linear density. Portable films in device-rich environments can be misleading because ECG leads, compression devices, and external hardware may project over the hilum [5]. While a two-view chest radiograph or computed tomography (CT) can clarify confusing projections and differentiate artifacts from true airway material [7,11,12], clinical judgment in this case prioritized immediate intervention. Given the patient’s clear history of home tracheostomy brush use and the distinct metallic appearance of the density, the team opted to bypass additional radiation and proceed directly to bronchoscopy, which serves as both the diagnostic gold standard and the therapeutic solution [1,2].

Iatrogenic foreign bodies constitute a meaningful subset of adult cases. Published reports specifically describe aspiration of tracheostomy components that appear as thin metallic lines on radiographs [3,4]. The daughter’s history provided a plausible mechanism and highlights the importance of standardized tracheostomy care education [15]. Finally, pleural effusion and basilar atelectasis can coexist with foreign bodies. In this patient, the effusion was likely multifactorial (heart failure history and post-operative status) rather than solely a direct sequela of the foreign body, but its presence necessitated careful evaluation [8-10].

## Conclusions

The right perihilar radiopaque density in this tracheostomized patient was not an artifact. Flexible bronchoscopy via the tracheostomy retrieved a tracheostomy cleaning brush from the right bronchus intermedius, with no complications and clinical stability post-removal. This case demonstrates that equivocal portable chest radiographs in device-rich settings should still prompt definitive airway evaluation when suspicion persists. CT may assist with localization, but bronchoscopy provides both diagnosis and therapy. Recognizing tracheostomy-care equipment as a potential iatrogenic foreign body can prevent delays and procedure-related complications.
